# Association of genetic variants related to plasma fatty acids with type 2 diabetes mellitus and glycaemic traits: a Mendelian randomisation study

**DOI:** 10.1007/s00125-019-05019-0

**Published:** 2019-11-05

**Authors:** Shuai Yuan, Susanna C. Larsson

**Affiliations:** 1 Department of Surgical Sciences, Uppsala University, Uppsala, Sweden; 2 Unit of Cardiovascular and Nutritional Epidemiology, Institute of Environmental Medicine, Karolinska Institutet, Nobelsväg 13, 17177 Stockholm, Sweden

**Keywords:** Fatty acids, Genetic variants, Glycaemic traits, Mendelian randomisation, Type 2 diabetes

## Abstract

**Aims/hypothesis:**

Epidemiological data on the associations of circulating fatty acid levels with type 2 diabetes are inconsistent. We conducted a two-sample Mendelian randomisation study to explore the causal associations of plasma levels of ten fatty acids with type 2 diabetes and glycaemic traits.

**Methods:**

Thirteen SNPs associated with circulating levels of ten individual fatty acids at the genome-wide significance level (*p* < 5 × 10^−8^) were selected as instrumental variables for the exposures. For the outcomes, summary-level data were obtained from the DIAbetes Genetics Replication And Meta-analysis (DIAGRAM) consortium for type 2 diabetes (898,130 individuals) and from the Meta-Analyses of Glucose and Insulin-related traits Consortium (MAGIC) for the glycaemic traits (up to 46,186 non-diabetic individuals). The inverse-variance weighted method was used for analyses.

**Results:**

Genetic predisposition to higher plasma levels of eight of the ten fatty acids were statistically significantly associated with lower or higher odds of type 2 diabetes. The OR per one SD increment of each fatty acid was 0.93 (95% CI 0.90, 0.96; *p* = 2.21 × 10^−5^) for α-linolenic acid, 0.96 (95% CI 0.94, 0.98; *p* = 1.85 × 10^−4^) for linoleic acid, 0.86 (95% CI 0.81, 0.91; *p* = 6.68 × 10^−7^) for palmitoleic acid, 0.87 (95% CI 0.81, 0.93; *p* = 2.21 × 10^−5^) for oleic acid, 1.08 (95% CI 1.03, 1.12; *p* = 0.002) for eicosapentaenoic acid, 1.04 (95% CI 1.02, 1.07; *p* = 0.001) for docosapentaenoic acid, 1.03 (95% CI 1.02, 1.05; *p* = 2.51 × 10^−5^) for arachidonic acid and 1.09 (95% CI 1.03, 1.15; *p* = 0.003) for stearic acid. The same eight fatty acids were also associated with fasting glucose levels and HOMA-B. The associations, except that for palmitoleic acid, were driven by variants in *FADS1/2*.

**Conclusions/interpretation:**

Genetic predisposition to higher circulating levels of eight out of ten fatty acids was associated with type 2 diabetes, fasting glucose and islet beta cell function. However, the associations, except that for palmitoleic acid, were driven by variants in *FADS1/2*, which encode enzymes with a key role in fatty acid metabolism.

**Electronic supplementary material:**

The online version of this article (10.1007/s00125-019-05019-0) contains peer-reviewed but unedited supplementary material, which is available to authorised users.

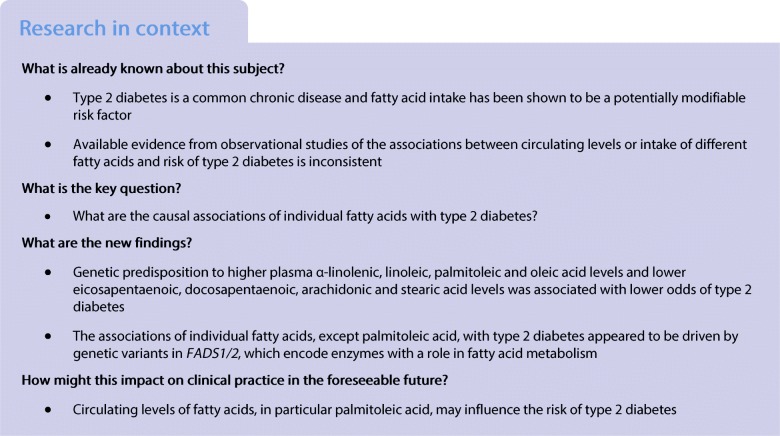



## Introduction

Type 2 diabetes mellitus is a common chronic disease and its prevalence has quadrupled in the past three decades globally [1]. It is estimated that around 8–9% of adults had type 2 diabetes in 2015 [1, 2]. Affected individuals have high morbidity risk and mortality risk due to complications such as cardiovascular and kidney diseases, which impose a huge burden on medical systems and society [3].

**Fig. 1:**
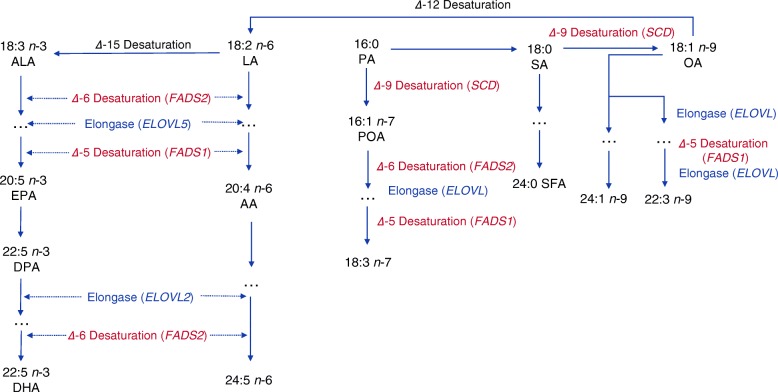
The role of enzymes encoded by *FADS*, *SCD* and *ELOVL* in the metabolism of fatty acids. Desaturation enzymes are shown in red and elongases in blue

Among the major driving factors of the global type 2 diabetes epidemic, dietary fatty acid intake has been shown to be a potentially modifiable risk factor for type 2 diabetes [4]. The concentration of different fatty acids in plasma and erythrocytes partly reflects dietary intake but is also determined by endogenous fatty acid production mediated by various fatty acid desaturases encoded by the *FADS1*, *FADS2* and *SCD* genes as well as various fatty acid elongases encoded by different *ELOVL* genes (Fig. 1) [5].

Available evidence from observational studies of the associations of circulating levels or intake of *n*-3 polyunsaturated fatty acids (PUFAs) [6, 7], *n*-6 PUFAs [8, 9] and saturated fatty acids (SFAs) [10, 11] with risk of type 2 diabetes are inconsistent. Data on monounsaturated fatty acids (MUFAs) in relation to type 2 diabetes are scarce. The inconsistent findings may partly be related to measurement errors in the assessment of dietary fatty acid intake, which could bias the true association between a categorical exposure and outcome in any direction [12]. The disagreements may also be due to residual confounding introduced by certain health behaviours or nutrients that are correlated with fatty acid levels or intake and also related to type 2 diabetes risk [4].

**Fig. 2:**
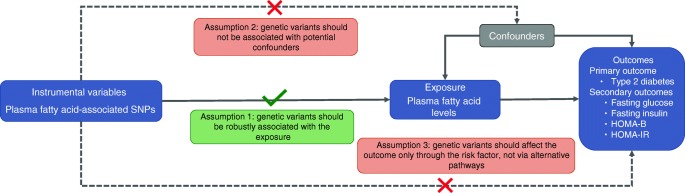
Schematic diagram of the MR assumptions underpinning an MR analysis of the association of plasma fatty acid levels with type 2 diabetes, fasting glucose, fasting insulin, HOMA-B and HOMA-IR. The dashed lines represent potential causal associations between variables that would represent violations of the MR assumption

Mendelian randomisation (MR) studies can strengthen the causal inference on exposure–outcome relationships by using genetic variants as instrumental variables for an exposure [13]. This method diminishes confounding since genetic variants (alleles) are randomly assorted at meiosis, thereby having no connection to self-selected lifestyle factors and behaviours [13]. It also overcomes reverse causality, a potential drawback in observational studies, as allelic randomisation precedes the onset of disease [13]. There are three assumptions for an MR study design (Fig. 2): (1) the genetic variants selected as instrumental variables should be robustly associated with the exposure; (2) the used instrumental variables should not be associated with any potential confounders; (3) the genetic variants of an exposure should affect the risk of the outcome merely through the risk factor, not via other alternative pathways [14].

We conducted a two-sample MR study to explore the causal associations of ten major fatty acid plasma levels and type 2 diabetes. As complementary analyses, we assessed whether the ten fatty acids were associated with fasting glucose and insulin levels, islet beta cell function and insulin resistance. HOMA-B and HOMA-IR were used as proxies of the islet beta cell function and insulin resistance, respectively.

## Methods

### Study design

An overview of the study design is displayed in Fig. 2. The present two-sample MR study is based on publicly available summary-level data on genetic associations with ten fatty acids, type 2 diabetes and glycaemic traits from genome-wide association studies (GWASs) (electronic supplementary material [ESM] Table 1). In all underlying studies included in the GWASs, the genetic association estimates were adjusted for age, sex and study-specific covariates. All these studies had been approved by a relevant institutional review board and participants had provided informed consents. The present MR study was approved by the Swedish Ethical Review Authority.

### SNP selection

Summary-level data of fatty acid levels were obtained from the hitherto largest GWASs of plasma phospholipid fatty acids or total plasma fatty acids (ESM Table 1) [15–17]. The ten fatty acids included in this MR study were α-linolenic acid (ALA), eicosapentaenoic acid (EPA), docosapentaenoic acid (DPA), docosahexaenoic acid (DHA), linoleic acid (LA), arachidonic acid (AA), palmitoleic acid (POA), oleic acid (OA), palmitic acid (PA) and stearic acid (SA). Fifteen SNPs associated with one or more of the fatty acids were selected at the genome-wide significance level (*p* < 5 × 10^−8^) (Table 1). Two SNPs (rs780093 and rs780094) were located in the *GCKR* locus, which is a highly pleiotropic locus associated with a number of phenotypes, such as lipids, BMI, alcohol intake and serum calcium levels [18]. In particular, those two SNPs were more strongly associated with type 2 diabetes and glycaemic traits than with plasma DPA and POA levels (Table 1 and ESM Table 2) and were therefore excluded from all analyses. Detailed information on the SNPs is displayed in Table 1 and ESM Table 2. SNPs associated with *n*-3 and *n*-6 PUFAs were identified in GWASs of 8866 and 8631 individuals of European ancestry, respectively [16, 17]. SNPs associated with *n*-7 MUFAs, *n*-9 MUFAs and SFAs were identified from five prospective studies with 8961 individuals of European descent [15]. For each fatty acid, all selected SNPs were in different gene regions and linkage equilibrium (i.e. uncorrelated). However, SNPs located in or close to genes (*FADS1, FADS2*, *SCD* and *ELOVL2*) that encode enzymes with a central role in the metabolic pathway of fatty acids (Fig. 1) were associated with multiple fatty acids. Since the percentage of each fatty acid among the total plasma fatty acids varies largely, we used 1 SD as the unit of change of individual fatty acid level [19]. One SD change in the present study corresponded to 2.69, 1.96, 0.05, 0.30, 0.17, 0.89, 1.17, 0.18, 1.64 and 1.19 percentage units of total fatty acid for LA, AA, ALA, EPA, DPA, DHA, OA, POA, PA and SA, respectively [19].

**Table 1:** Characteristics of the SNPs associated with fatty acids and their associations with type 2 diabetes

Type of FA	FA	SNP	Chr	Nearby gene	EA	% variance explained	Association with fatty acids			Association with type 2 diabetes		
							β^a^	SE	*p* value	β^b^	SE	*p* value
*n*-3 PUFA	ALA	rs174547	11	*FADS1*	C	1.0	0.02	0.001	3.50 × 10^−64^	−0.028	0.007	2.70 × 10^−5^
*n*-3 PUFA	EPA	rs3798713	6	*ELOVL2*	C	0.4	0.04	0.005	1.90 × 10^−12^	−0.005	0.006	0.45
*n*-3 PUFA	EPA	rs174538	11	*FADS1*	G	1.7	0.08	0.005	5.40 × 10^−58^	0.026	0.007	1.40 × 10^−4^
*n*-3 PUFA	DPA	rs780094^c^	2	*GCKR*	T	0.5	0.02	0.003	9.00 × 10^−9^	−0.065	0.007	1.60 × 10^−23^
*n*-3 PUFA	DPA	rs3734398	6	*ELOVL2*	C	2.7	0.04	0.003	9.70 × 10^−43^	−0.006	0.006	0.37
*n*-3 PUFA	DPA	rs174547	11	*FADS1*	T	8.4	0.08	0.003	3.80 × 10^−154^	0.028	0.007	2.70 × 10^−5^
*n*-3 PUFA	DHA	rs2236212	6	*ELOVL2*	G	0.7	0.11	0.014	1.30 × 10^−15^	0.005	0.006	0.46
*n*-6 PUFA	LA	rs10740118	10	*JMJD1C*	G	0.2–0.7	0.25	0.050	8.10 × 10^−9^	0.013	0.006	0.04
*n*-6 PUFA	LA	rs174547	11	*FADS1*	C	7.6–18.1	1.47	0.050	5.00 × 10^−274^	−0.028	0.007	2.70 × 10^−5^
*n*-6 PUFA	LA	rs16966952	16	*NTAN1*	G	0.5–2.5	0.35	0.040	1.20 × 10^−15^	0.000	0.007	0.99
*n*-6 PUFA	AA	rs174547	11	*FADS1*	T	3.7–37.6	1.69	0.020	3.3 × 10^−971^	0.028	0.007	2.70 × 10^−5^
*n*-6 PUFA	AA	rs16966952	16	*NTAN1*	G	0.1–0.6	0.20	0.030	2.40 × 10^−10^	0.000	0.007	0.99
*n*-7 PUFA	POA	rs780093^c^	2	*GCKR*	T	0.2–0.9	0.02	0.003	9.80 × 10^−10^	−0.065	0.007	4.60 × 10^−23^
*n*-7 PUFA	POA	rs6722456	2	*RN7SKP93*	G	0.01–0.6	0.05	0.009	4.10 × 10^−8^	−0.021	0.022	0.36
*n*-7 PUFA	POA	rs603424	10	*SCD/PKD2L1*	G	0.3–1.6	0.03	0.004	5.70 × 10^−15^	−0.017	0.009	0.05
*n*-7 PUFA	POA	rs11190604	10	*HIF1AN*	G	0.02–0.7	0.02	0.004	5.70 × 10^−9^	−0.020	0.008	0.01
*n*-7 PUFA	POA	rs102275	11	*FADS1/2*	C	0.15–1.0	0.02	0.003	6.60 × 10^−13^	−0.028	0.007	2.00 × 10^−5^
*n*-9 PUFA	OA	rs102275	11	*FADS1/2*	C	0.3–2.1	0.23	0.020	2.20 × 10^−32^	−0.028	0.007	2.00 × 10^−5^
SFA	PA	rs2391388	1	*ALG14*	C	0.2–1.0	0.18	0.030	2.70 × 10^−11^	−0.003	0.006	0.66
SFA	SA	rs6675668	1	*ALG14*	G	0.4–1.4	0.17	0.020	2.20 × 10^−18^	0.005	0.006	0.42
SFA	SA	rs11119805	1	*LPGAT1*	T	0.01–0.7	0.17	0.030	2.80 × 10^−9^	−0.008	0.010	0.43
SFA	SA	rs102275	11	*FADS1/2*	T	0.3–1.2	0.18	0.020	1.30 × 10^−20^	0.028	0.007	2.00 × 10^−5^

^a^The β coefficients represent the change in percentage of total fatty acids for each additional effect allele

^b^The β coefficients represent the log_10_ OR of type 2 diabetes for each additional effect allele

^c^SNPs in the *GCKR* gene were excluded from all analyses due to multiple pleiotropic associations with potential confounders

Chr, chromosome; EA, effect allele; FA, fatty acid

### Outcome sources

Summary-level data for the association of fatty-acid-associated SNPs with type 2 diabetes were obtained from the DIAbetes Genetics Replication And Meta-analysis (DIAGRAM) consortium, a publicly available GWAS of 32 studies with a total of 898,130 individuals (74,124 with type 2 diabetes and 824,006 without) of European ancestry (ESM Table 1) [20]. We used the type 2 diabetes data without BMI adjustment in our primary analyses, and then performed a sensitivity analysis by using BMI-adjusted data.

Summary-level data for the fatty-acid-related SNPs with fasting glucose, fasting insulin, HOMA-B and HOMA-IR were obtained from the Meta-Analyses of Glucose and Insulin-related traits Consortium (MAGIC). Fasting glucose data was based on a meta-analysis of 21 GWASs including 46,186 non-diabetic individuals of European ancestry [21]. Summary-level data of fasting insulin, HOMA-B and HOMA-IR were acquired from meta-analysis of 20 GWASs with, respectively, 38,238, 36,466 and 37,037 non-diabetic individuals of European descent [21].

### Statistical analyses

The fixed-effects inverse-variance weighted method was used to assess the associations of plasma phospholipid fatty acid levels with the outcomes. The OR of type 2 diabetes was calculated per 1 SD increment in genetically predicted plasma fatty acid levels. For fasting glucose and insulin, HOMA-B and HOMA-IR, β estimates were scaled per 1 SD increment in plasma fatty acid levels. Sensitivity analyses based on the random-effect inverse-variance weighted method and the weighted median method [22] were performed where more than three SNPs were available as instrumental variables. This was the case only for POA. All *p* values were two-sided. Associations with *p* values below 0.005 were deemed statistically significant after Bonferroni correcting for ten fatty acids. A *p* value between 0.001 and 0.05 was regarded as suggestive evidence of an association. The statistical analyses were performed in Stata/SE 15.0 (StataCorp, College Station, TX, USA) by using the mrrobust package [23].

## Results

### Fatty acids and type 2 diabetes

**Fig. 3:**
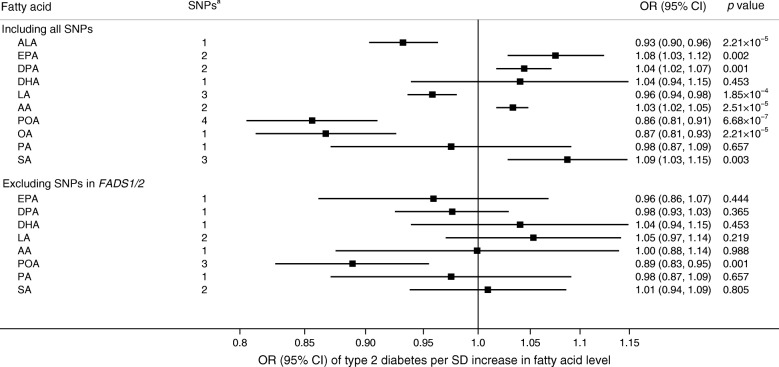
Associations between plasma fatty acid levels and type 2 diabetes from MR analyses. ^a^Number of SNPs included in the analysis of each fatty acid

**Fig. 4:**
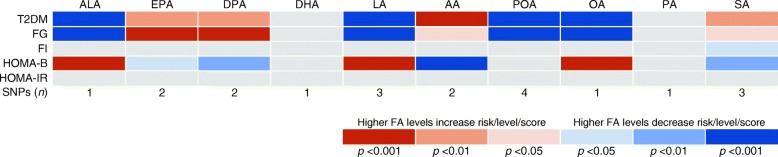
MR associations of plasma fatty acid levels with type 2 diabetes, fasting glucose, fasting insulin, HOMA-B and HOMA-IR. FA, fatty acid; FG, fasting glucose; FI, fasting insulin; T2DM, type 2 diabetes

Genetic predisposition to higher levels of eight of the ten fatty acids was significantly associated with lower or higher odds of type 2 diabetes in analyses based on all SNPs (Figs 3, 4). The OR per 1 SD increase in plasma levels of each fatty acid was 0.93 (95% CI 0.90, 0.96; *p* = 2.21 × 10^−5^) for ALA, 0.96 (95% CI 0.94, 0.98; *p* = 1.85 × 10^−4^) for LA, 0.86 (95% CI 0.81, 0.91; *p* = 6.68 × 10^−7^) for POA, 0.87 (95% CI 0.81, 0.93; *p* = 2.21 × 10^−5^) for OA, 1.08 (95% CI 1.03, 1.12; *p* = 0.002) for EPA, 1.04 (95% CI 1.02, 1.07; *p* = 0.001) for DPA, 1.03 (95% CI 1.02, 1.05; *p* = 2.51 × 10^−5^) for AA and 1.09 (95% CI 1.03, 1.15; *p* = 0.003) for SA (Fig. 3). Only the association with POA remained after exclusion of SNPs (rs174547 and rs102275) in or close to *FADS1/2*, which generally explained the major proportion of variance in plasma fatty acid levels (Table 1). The minor allele (C) of both rs174547 and rs102275 was associated with higher plasma ALA, LA, POA and OA levels and lower plasma DPA, AA and SA levels as well as with lower odds of type 2 diabetes (Table 1). The SNPs in the *FADS1/2* gene cluster had no significant pleiotropic associations with potential confounders (ESM Table 3). SNPs in *ELOVL2* were associated with plasma levels of all three long-chain *n*-3 PUFAs (EPA, DPA and DHA) but were not associated with type 2 diabetes (Table 1) and had no significant pleiotropic associations with any other traits (ESM Table 3). The association between genetic predisposition to higher POA levels and lower odds of type 2 diabetes remained in analyses based on the random-effects inverse-variance weighted method and the weighted median method (ESM Fig. 1). No significant differences between the results based on type 2 diabetes data without and with adjustment for BMI were observed (ESM Fig. 2).

### Fatty acids and glycaemic traits

Genetic predisposition to higher plasma ALA, LA, POA and OA levels were associated with lower fasting glucose levels and higher HOMA-B score, whereas genetic predisposition to higher plasma AA, EPA, DPA and SA levels were associated with higher fasting glucose levels and lower HOMA-B score (Fig. 4 and ESM Table 4). These associations seemed to be driven by SNPs in or close to *FADS1/2* (ESM Table 2). None of the fatty acids was associated with fasting insulin levels or HOMA-IR (Fig. 4 and ESM Table 4).

## Discussion

The present MR study found evidence that genetic predisposition to higher plasma levels of ALA, LA, POA and OA were associated with lower odds of type 2 diabetes, lower fasting glucose and higher HOMA-B score, whereas genetic predisposition to higher plasma levels of EPA, DPA, AA and SA were associated with higher odds of type 2 diabetes, higher fasting glucose and lower HOMA-B score. The associations except that for POA were driven by variants in *FADS1/2*, which explained the major proportion of variance in plasma fatty acid levels.

A major limitation of this MR study is that SNPs in the *FADS1/2* gene cluster were associated with most fatty acids and only three or fewer SNPs were available as instrumental variables for individual fatty acids, except for POA. This limited the possibility for disentangling the association of individual fatty acids with type 2 diabetes, and performing sensitivity analyses to explore pleiotropy. The strong associations of *FADS1/2* with fatty acid levels are not surprising because *FADS1* and *FADS2* encode Δ-5 desaturase and Δ-6 desaturase, respectively, which are key rate-limiting enzymes in the metabolism of fatty acids (Fig. 1). Strong correlations between circulating biomarkers is not only an issue in the present MR study but can also be an issue in for example MR studies on metabolomics where several metabolites can be highly correlated.

Our results are broadly in agreement with those of previous smaller studies of the associations of *FADS1* and *FADS2* genetic variants or Δ-5 and Δ-6 desaturase activities with type 2 diabetes. A Chinese case–control study, including 331 individuals with type 2 diabetes and 421 healthy control individuals, found that the minor allele (T) of rs174616 in the *FADS1-FADS2* gene cluster was associated with decreased AA/LA ratio and lower odds of type 2 diabetes [24]. Results from a nested case-cohort study of 2653 German adults showed no overall association of rs174546, which is in complete linkage disequilibrium with rs174547, with type 2 diabetes [25]. However, that study revealed that a higher proportion of LA in erythrocytes and lower proportions of γ-linolenic acid and dihomo-γ-linolenic acid were associated with lower risk of type 2 diabetes. Furthermore, fatty acid ratios that reflect Δ-5 desaturase and Δ-6 desaturase activity were respectively inversely and positively associated with type 2 diabetes [25]. Similarly, results from a Finnish cohort study (407 overweight adults) [26] and an Iranian case–control study (95 individuals with type 2 diabetes and 95 without) [27] showed that Δ-5 desaturase activity was inversely associated with type 2 diabetes. Finally, a cross-sectional study of 576 Korean men showed that variants in the *FADS* gene cluster were associated with the proportions of LA, dihomo-γ-linolenic acid, and AA in serum phospholipids as well as with fasting insulin levels and HOMA-IR [28].

Available data on the associations of circulating POA levels with the risk of type 2 diabetes and glycaemic traits are inconsistent [29–33]. Several population-based observational studies found that increased plasma or erythrocyte membrane POA level was associated with higher blood glucose levels [29], insulin resistance [30] and incidence of diabetes [31]. However, in a prospective longitudinal study including 3630 Americans, circulating level of POA was not associated with the risk of diabetes and the positive association between POA level and insulin resistance was merely observed among men [30]. In addition, several studies concluded that increasing plasma POA level lowered fasting insulin, insulin resistance and the risk of type 2 diabetes [32]; this has been verified by cell experimental studies [33]. In the present study, genetic evidence, excluding the influences from *FADS* and *GCKR*, supports a protective effect of increasing POA levels on fasting glucose level and type 2 diabetes. Discrepancy between these findings may be attributable to residual confounding commonly affecting observational studies, such as type 2 diabetes-related nutrients, lifestyle determinants or endogenous sources introduced by dietary POA intake or de novo lipogenesis (e.g. key enzymes in fatty acid metabolism, inflammatory factors and metabolic traits encoded by POA synthesis-related genes).

As for potential biological mechanisms, the associations of plasma fatty acid levels and genetic variations in *FADS1/2* with type 2 diabetes may be mediated by impaired glucose tolerance and beta cell dysfunction, as suggested by the present MR study. It has been postulated that POA may prevent beta cell apoptosis induced by glucose or SFAs [34]. Fatty acids are crucial structural components of cell membranes. The flexibility of a membrane, determined by the ratio of unsaturated to saturated fatty acyl chains of its phospholipids, influences the effectiveness of glucose transport via insulin-dependent glucose transporters [35]. A shift from unsaturated towards saturated fatty acyl chains of membrane phospholipids results in a decrease in glucose effectiveness and insulin sensitivity [36]. Fatty acids are further precursors of eicosanoids, which may affect type 2 diabetes risk through inflammation [36, 37]. An experimental study in mice showed that loss of Δ-5 desaturase activity promoted hepatic inflammation and implied that endogenously synthesised AA and EPA are key determinants of inflammatory disease progression [38]. Associations of fatty acids with type 2 diabetes might also be explained by the effects of fatty acids on the gastrointestinal tract [37], intestinal microbiome [39] and nervous system [37].

A further mechanism whereby fatty acids might affect the risk of type 2 diabetes is through blood cholesterol levels, which are inversely associated with risk of type 2 diabetes [40]. A meta-analysis of 60 trials revealed that SFAs increase total cholesterol and LDL-cholesterol levels, whereas MUFAs and PUFAs have the opposite effect, and that all types of fatty acid increase HDL-cholesterol levels [41]. The minor allele (C) of both rs174547 and rs102275 is associated with decreased levels of LDL-cholesterol, HDL-cholesterol and total cholesterol [42]. Thus, we would have expected a higher rather than lower odds of type 2 diabetes for the minor allele of rs174547 and rs102275 if the associations were mediated by cholesterol levels.

### Conclusions

This MR study showed that genetic predisposition to higher plasma ALA, LA, POA and OA levels and lower EPA, DPA, AA and SA levels was associated with lower odds of type 2 diabetes, lower fasting glucose levels and higher HOMA-B score. However, the causality for any individual fatty acid, except POA, cannot be inferred from this study due to the high correlation between the fatty acids. Notably, the observed associations, except that for POA, appeared to be driven by genetic variants in or close to *FADS1/2*. Replication of our finding for POA in an independent MR study would provide strong evidence for a causal effect. Further research is needed to unravel the role of individual fatty acids in the prevention of type 2 diabetes.

## Electronic supplementary material

ESM Tables (XLSX 31 kb)

ESM Figures (PDF 295 kb)

## Data Availability

All data included in this study are available in the article and ESM.

## Abbreviations

AA: Arachidonic acid
ALA: α-Linolenic acid
DHA: Docosahexaenoic acid
DIAGRAM: DIAbetes Genetics Replication And Meta-analysis
DPA: Docosapentaenoic acid
EPA: Eicosapentaenoic acid
GWAS: Genome-wide association study
LA: Linoleic acid
MAGIC: Meta-Analyses of Glucose and Insulin-related traits Consortium
MR: Mendelian randomisation
MUFA: Monounsaturated fatty acid
OA: Oleic acid
PA: Palmitic acid
POA: Palmitoleic acid
PUFA: Polyunsaturated fatty acid
SA: Stearic acid
SFA: Saturated fatty acid
