# Supplementary material for: Association of genetic variants related to plasma fatty acids with type 2 diabetes mellitus and glycaemic traits: a Mendelian randomisation study
Source: Diabetologia. 2019 Nov 5;63(1):116–23. doi: 10.1007/s00125-019-05019-0 (PMC6890658; doi:10.1007/s00125-019-05019-0)
Supplement: Supplementary file 2 — (PDF 295 kb) [file 125_2019_5019_MOESM2_ESM.pdf]

Electronic Supplementary Figures

ESM Figure 1. Associations between palmitoleic acid and type 2 diabetes in Mendelian randomization analyses based on different methods

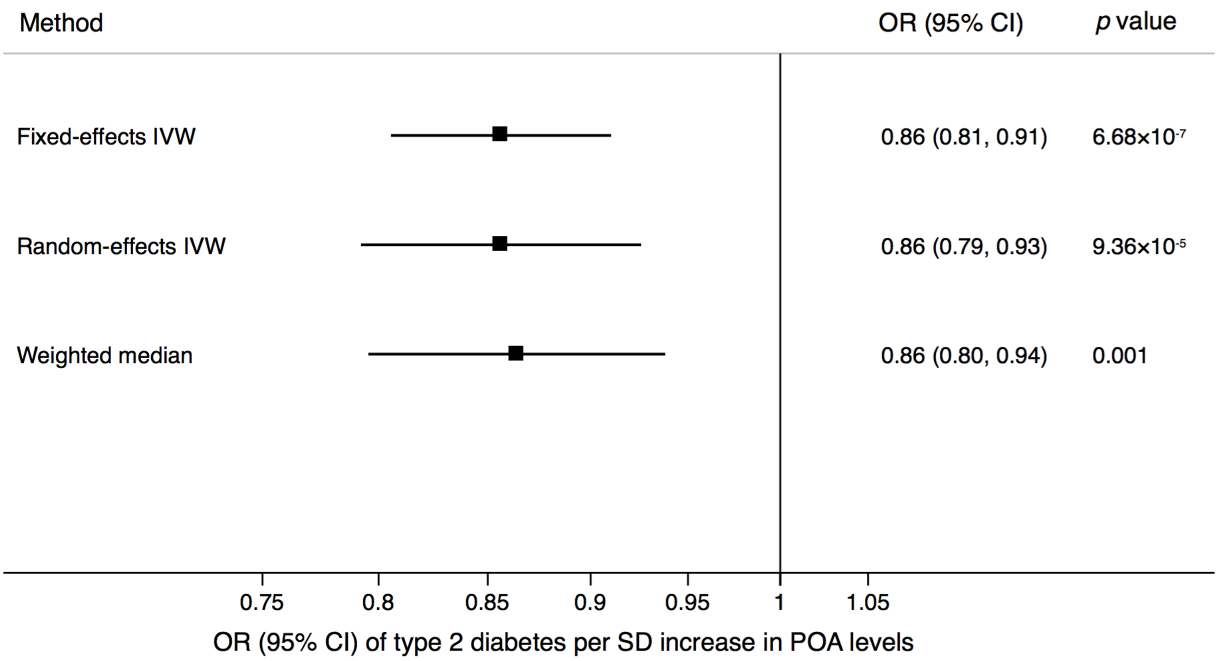

CI indicates confidence interval; IVW, inverse-variance weighted; OR, odds ratio; POA, palmitoleic acid.

ESM Figure 2. Associations between plasma fatty acid levels and body mass index-adjusted type 2 diabetes from Mendelian randomization analyses

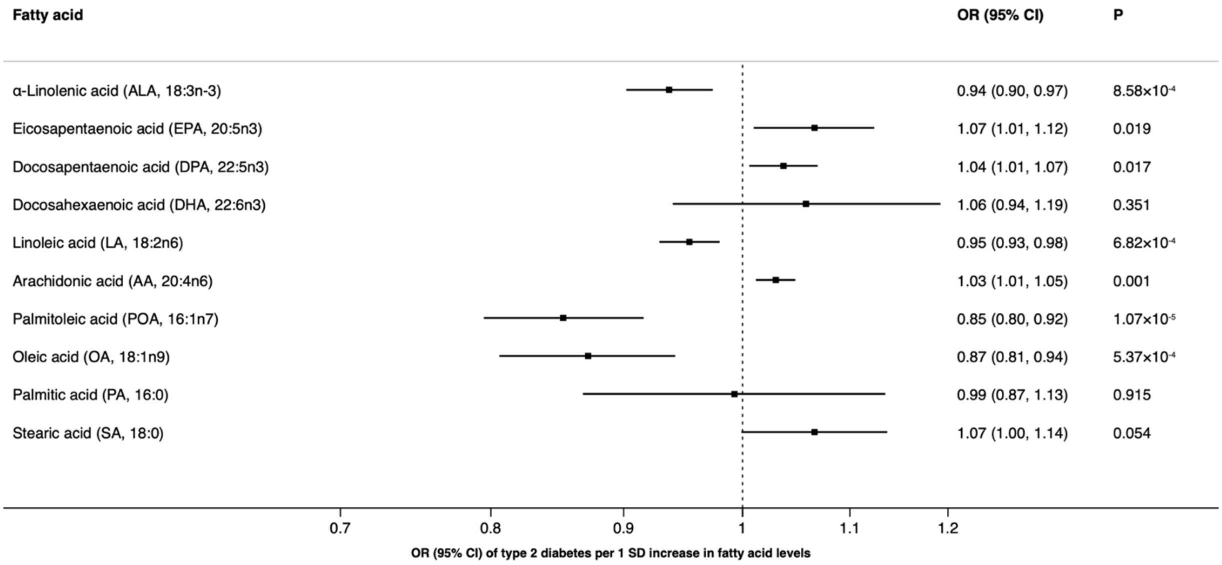

CI indicates confidence interval; OR, odds ratio; SD, standard deviation.

Electronic Supplementary Tables (Excel spreadsheets)

ESM Table 1. Details of studies and datasets used for analyses

ESM Table 2. Characteristics of the single-nucleotide polymorphisms associated with fatty acids and their associations with type 2 diabetes, fasting glucose, fasting insulin, β-cell function and insulin resistance

ESM Table 3. Pleiotropic associations of SNPs in the FADS1/2 gene cluster and phenotypes other than fatty acid levels

ESM Table 4. Associations of plasma plasma fatty acids with fasting glucose, fasting insulin, β-cell function and insulin resistance from Mendelian randomization
